# Responsive Fluorescent Coumarin–Cinnamic Acid Conjugates for α-Glucosidase Detection

**DOI:** 10.3389/fchem.2022.927624

**Published:** 2022-06-28

**Authors:** Dong Luo, Xin Zhang, Xiaoying Li, Yi-Yu Zhen, Xingyi Zeng, Zhuang Xiong, Yinghui Zhang, Hongguang Li

**Affiliations:** ^1^ School of Biotechnology and Health Science, Wuyi University, Jiangmen, China; ^2^ Guangdong Provincial Key Laboratory of Large Animal Models for Biomedicine, South China Institute of Large Animal Models for Biomedicine, Wuyi University, Jiangmen, China

**Keywords:** α-glucosidase, fluorescent probe, responsive probe, coumarin, environmentally sensitive dyes, cinnamic acid

## Abstract

α-Glucosidase is a potent drug target for treating type II diabetes mellitus. A great number of α-glucosidase inhibitors have been developed based on the molecular skeletons of bioactive natural products. However, efficient fluorescent probes for α-glucosidase detection are still limited, not to mention the probes with additional inhibitory functions. In this work, aiming for the enzyme’s highly specific detection, we designed and synthesized two environmentally sensitive fluorescent probes, namely, LD01 and LD02, respectively, based on conjugates of coumarin and cinnamic acid derivatives. We found a significant responsive emission enhancement upon LD02’s binding to α-glucosidase. These newly designed probes can act as a simple but efficient tool to evaluate the binding affinity of α-glucosidase to their inhibitors.

## Introduction

Diabetes mellitus (DM) can be categorized into three types (type I, type II, and gestational diabetes), with type II DM accounting for ninety percent of the total number of patients (Chen et al., 2015). Pathological features of type II DM are characterized by hyperglycemia arising from various causes such as a relative deficiency of insulin secretion, insulin resistance, or other malfunctioning factors on carbohydrate metabolism (Teng and Chen, 2017). Long-term high blood glucose levels in human bodies can lead to various complications, such as kidney disease, cataract, and vascular diseases (Prattichizzo et al., 2019). α-Glucosidase is a membrane-bound enzyme in small intestinal epithelial cells, playing a pivotal role in the digestion of carbohydrates by direct participation in the metabolic pathway of glycogen and starch (Tang et al., 2017). Biochemically, in the last step of glycolysis, it can breakdown the α-1,4 glycosidic bonds from the non-reducing saccharide chains’ end, hydrolyzing the long oligosaccharide chains into single glucose. Then the digested glucose can be absorbed by the small intestine and transported to the blood. When this physiological process overacts due to excessive ingestion of carbohydrates, hyperglycemia occurs, leading to the development of DM (Guo et al., 2021).

Because of the biological and pharmacological importance of α-glucosidase, a great number of natural products and synthetic compounds were developed for α-glucosidase-targeting inhibition (Liu and Ma, 2017; Dhameja and Gupta, 2019; Gutiérrez-González et al., 2021; Trang et al., 2021). However, efficient probes targeting α-glucosidase and the corresponding detection methods are still very rare. Up to now, only limited examples were reported in recent years about α-glucosidase’s molecular detection (Zhang et al., 2015; Ao et al., 2017; Kong et al., 2017; Li et al., 2018; Cheng et al., 2019), which are all focused on the catalytic function of α-glucosidase. Because this type of technical system involved multiple steps in the sensing protocol, the quantitative detection of α-glucosidase cannot be satisfactorily acquired.

Environmentally sensitive fluorescent probes are widely used for protein detection and imaging, whose responsive emissions are highly sensitive to the microenvironmental properties, such as polarity, pH, or viscosity of the surrounding medium where the dyes are located (Shvadchak et al., 2017; Dong et al., 2020; Zhou et al., 2020). Intramolecular charge transfer (ICT)-based donor-π-acceptor dyes were found to exhibit strong emission in non-polar environments but fail to do so in highly polar solvents (Dai et al., 2020; Pal. et al., 2020). Theoretically, the binding pocket of a specific target protein for such fluorescent probes may be more hydrophobic than that in aqueous solutions.

Coumarin and cinnamic acid are two types of typical natural products widely distributed in nature, harboring typical useful motifs in medicinal chemistry (Gaudino et al., 2016; Adisakwattana, 2017; Annunziata et al., 2020; Ruwizhi and Aderibigbe, 2020). Recently, Xu et al. reported that the conjugates of coumarin and cinnamic acid derivatives are potent inhibitors against α-glucosidase (Xu et al., 2019). In this work, we designed and synthesized two fluorescent conjugates of dialkyl-substituted coumarin and cinnamic acid, LD01 and LD02, featuring donor-π-acceptor structures as two environmentally sensitive probes for α-glucosidase-targeting detection. We hypothesized the binding pocket inside α-glucosidase may provide a relatively non-polar environment for the fluorescent probes so that intense fluorescence emission will be activated when bound to α-glucosidase. Comprehensive photophysical studies and enzyme inhibitory assays demonstrated newly designed fluorescent probe LD02 can act as a fluorescent indicator for α-glucosidase, which can also serve as a potential tool for evaluating the binding affinity between α-glucosidase and its inhibitors.

## Experimental

### Instruments and Reagents

All chemicals and solvents were obtained from commercial suppliers without further purification. α-Glucosidase from *Saccharomyces cerevisiae* (EC 3.2.1.20) was purchased from Sigma-Aldrich. p-Nitrophenyl-α-D-galactopyranoside (PNPG) was obtained from Abcam. Nuclear magnetic resonance (NMR) spectra of new compounds were recorded in DMSO-*d*_6_ using a Bruker Advance 500 MHz spectrometer. High-resolution mass spectrometry (HRMS) was measured on a Thermo Fisher Scientific Q Exactive Orbitrap mass spectrometer. UV–Vis absorption spectra were measured using an Hitachi 3,900 absorption spectrophotometer (Tokyo, Japan). Fluorescence spectra were recorded using an Edinburgh FLS1000 fluorescence spectrophotometer (Edinburgh, UK).

### Synthesis of LD01 and LD02

The substituted 3-acetyl-aminocoumarin derivatives (1.0 mmol) were added to a solution of hydroxylamine hydrochloride (3.0 mmol) and pyridine (0.04 mmol) in ethanol (10.0 ml), and the mixture was stirred for 20 h at room temperature. When the reaction ended, the mixture was filtered to obtain precipitate which was then washed with ethanol to generate the substituted coumarin-based oxime product. DCC (1.1 mmol) and DMAP (0.2 mmol) were added to the solution of the substituted cinnamic acid at 0°C, and the mixture was stirred for 15 min; subsequently, the coumarin-based oxime compounds were added into the mixture and thoroughly stirred for another 12 h at room temperature. The mixture was quenched with water and extracted three times with DCM, washed with brine, and dried with magnesium sulfate. The crude product could be obtained under vacuum, and then purified by column chromatography to give the title product.

Compound LD01. Orange, sold; yield 70%; m.p. 196–198°C; ^1^H NMR (500 MHz, DMSO-*d*_6_) δ 7.86 (s, 1H), 7.64 (d, J = 8.6 Hz, 2H), 7.60 – 7.57 (m, 3H), 7.49 (d, J = 8.8 Hz, 1H), 6.69 (dd, J = 8.9, 2.5 Hz, 1H), 6.56 (d, J = 16.0 Hz, 1H), 6.52 (d, J = 2.6 Hz, 1H), 3.42 (q, J = 7.0 Hz, 4H), 2.05 (s, 3H), 1.11 (t, J = 7.0 Hz, 6H);^13^C NMR (126 MHz, DMSO-*d*_6_) δ 167.89, 160.51, 156.73, 152.69, 151.18, 143.08, 141.91, 133.99, 132.31, 130.61, 130.44, 123.99, 120.58, 117.64, 109.66, 108.03, 96.61, 44.57, 14.10, 12.77; HRMS (+ESI) [M + H]^+^ calcd. For C_24_H_23_BrN_2_O_4_: 483.0930; found: 483.0967.

Compound LD02. Yellow, sold; yield 65%; m.p. 167–168°C; ^1^H NMR (500 MHz, DMSO-*d*_6_) δ 8.63 (s, 1H), 7.93 (dd, J = 7.8, 1.6 Hz, 1H), 7.73 (ddd, J = 8.7, 7.3, 1.7 Hz, 1H), 7.46 (dt, J = 10.6, 6.8 Hz, 4H), 7.40 (td, J = 7.6, 1.1 Hz, 1H), 6.67 (d, J = 8.9 Hz, 2H), 6.20 (d, J = 15.9 Hz, 1H), 2.95 (s, 6H), 2.58 (s, 3H); ^13^C NMR (126 MHz, DMSO-*d*_6_) δ 195.51, 168.67, 158.90, 155.06, 152.02, 147.52, 145.12, 134.92, 131.24, 130.16, 125.37, 124.81, 121.99, 118.61, 116.56, 113.35, 112.20, 40.14, 30.52; HRMS (+ESI) [M + H]^+^ calcd. For C_22_H_20_N_2_O_4_: 377.1509; found: 377.1533.

### Photophysical Properties’ Measurements

All solvents used for spectroscopic studies are spectrum pure grade. The stock solution of each sample was prepared in DMSO (1 mM), and the working solution was obtained by diluting 30 μL of stock solutions into 3 ml of PBS buffer (10 mM, pH = 7.4) or other solvents in a quartz cuvette (1 cm × 1 cm). The absorption and emission spectra were measured at room temperature. The widths of the slits for both excitation and emission were 2 nm for all experiments.

### pH-Dependent Emission

The emission spectra of the probes in PBS buffer (pH from 3.0 to 8.0) were determined at room temperature. The pKa value was calculated using the Henderson–Hosslbalch equation (Finikova et al., 2003):

$$pH\;=\;pKa\;+\;\mathrm{lg}\;\left(\frac{\left[\text{A}\right]}{\left[\text{HA}\right]}\right).$$

### Fluorescence Titration of The Probes With α-Glucosidase

α-Glucosidase protein was titrated to the solution of the testing probe (LD01 or LD02) (10 μM), and the fluorescence emission spectra were recorded from 380 to 700 nm under excitation of 365 nm. The binding constants of Ka were obtained from a double logarithm regression curve (Wei et al., 2010):

1g[(I−Io)/Io] = 1gKa + n 1g[G],

where I and I_0_ are the current and initial fluorescence intensity, respectively, *K*_*a*_ is the binding constant, n is the number of binding sites for each α-glucosidase, and [G] is the total concentration of the α-glucosidase protein.

### Fluorescence Competitive Binding Assay

The binding complexes of α-glucosidase with LD02 were prepared by adding α-glucosidase (final concentration = 3.5 μM) to the solution of LD02 (10 μM) in 10 mM PBS buffer (pH = 7.4). The dissociation constant of a competitor to α-glucosidase can be determined by titrating it against binding complexes of α-glucosidase with LD02, and can be calculated according to the Cheng–Prusoff equation (Cheng and Prusoff, 1973).

### α-Glucosidase Inhibition Efficiency Test of LD01 and LD02

The inhibition efficiency of LD01 and LD02 were determined according to the procedures Xu et al. reported previously (Xu et al., 2019). Stock solutions of testing compounds were prepared in DMSO. Enzyme solution of α-glucosidase (2 U/mL) was prepared in 0.1 M PBS buffer. Nitrophenol-α-D-glucoside (PNPG) was dissolved in 0.1 M PBS buffer (pH 6.8) to obtain the substrate solution (4 mM). In total, 10 μL of α-glucosidase (final concentration 0.1 U/mL), 130 μL of PBS buffer, and 10 μL of the sample solution were added into 96-well plates in succession and incubated at 37°C for 10 min. Then 50 μL of substrate solution (PNPG final concentration 1 mmol/L) was added, followed by incubation at 37°C for another 30 min. The enzymatic activity was quantified by measuring the absorbance of the reaction mixture at 405 nm using a multimodel reader. The inhibition rate was calculated using the following equation:

$$Inhibition\;rate\;\left(\%\right)\;=\;\left[\left(A_{1}-A_{0}\right)/A_{0}\right]\;\times \;100\%,$$

where A_1_ and A_0_ are the absorbance values of testing samples and blank control, respectively. The IC_50_ value was obtained from a plot of the inhibition rate *versus* the sample concentration curve. The assays were carried out in triplicate and the mean values were given.

## Result and Discussion

### Synthesis and Characterization of LD01 and LD02

**SCHEME 1:**
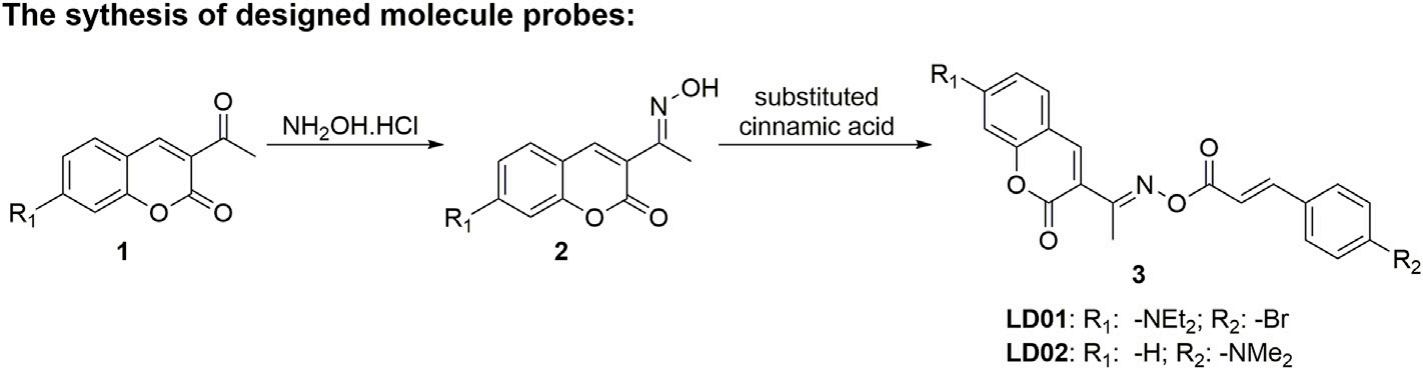
Synthetic route of LD01 and LD02.

LD01 and LD02 could be obtained through a two-step synthesis as shown in Scheme 1. In brief, substituted 3-acetylcoumarin derivatives reacted with hydroxylamine hydrochloride under the basic condition to give the substituted coumarin-based oxime product, followed by acylation with corresponding cinnamic acid to produce the designed molecular probes **LD01** and **LD02**, which were well characterized by ^1^H NMR, ^13^C NMR, and HRMS (supporting information, Supplementary Figures S1–6).

### Photophysical Properties’ Studies

Absorption spectra of LD01 and LD02 were measured at room temperature in PBS buffer (pH = 7.4). LD01 has two absorption bands at 410 nm (*ε* = 14,800 M cm^−1^) and 290 nm (*ε* = 9800 M cm^−1^) assigned to π → π* transitions from diethylamino-coumarin and bromo cinnamic ester moiety, respectively. LD02 has an absorption band at around 365 nm (*ε* = 4900 M cm^−1^) resulting from the mixed π → π* transitions of coumarin and cinnamic ester moieties. For the emission spectra, LD01 and LD02 show one emission band with maximum emission at 500 and 457 nm, respectively, while exhibiting very low emission quantum yield, 5.3% for LD01, and 3.2% for LD02 in PBS buffer (pH = 7.4). It has been well documented that dialkyl amine-substituted coumarin or cinnamic acid derivatives are ICT-based dyes, and their emission band or intensity is affected by the surrounding polarity. We first determined the emission spectra of LD01 and LD02 in various solvents of different polarities, such as water, methanol, acetonitrile, N,N-dimethylformamide, and dioxane (Figures 2A–D). The emission intensity of LD01 accrues in an increasing order of polarity. LD02 shows a similar tendency except for the emission of dioxane, which may result from the poor solubility of LD02 in it. Moreover, the emission band of LD02 shows a 40-nm blue shift in dioxane as compared to that in an aqueous buffer. We then measured the fluorescence of LD01 and LD02 in solvents with water and dioxane mixed at different ratios, representing different degrees of solvent polarity. As shown in Figures 2E–H, in the mixed solvent system, both LD01 and LD02’s fluorescent emission shows a similar tendency in response to polarity change from different solvents, which suggests both LD01 and LD02 can be potentially used to detect α-glucosidase *via* responsive emission. In the meantime, the pH effect on the emission of LD01 and LD02 was investigated. The emission spectra of LD01 and LD02 are less sensitive to pH variation (Figure 3) from 3.0 to 8.0 than the polarity changes; the pKa values of LD01 and LD02 were worked out to be 4.2 and 4.6 for LD01 and LD02, respectively.

**FIGURE 1:**
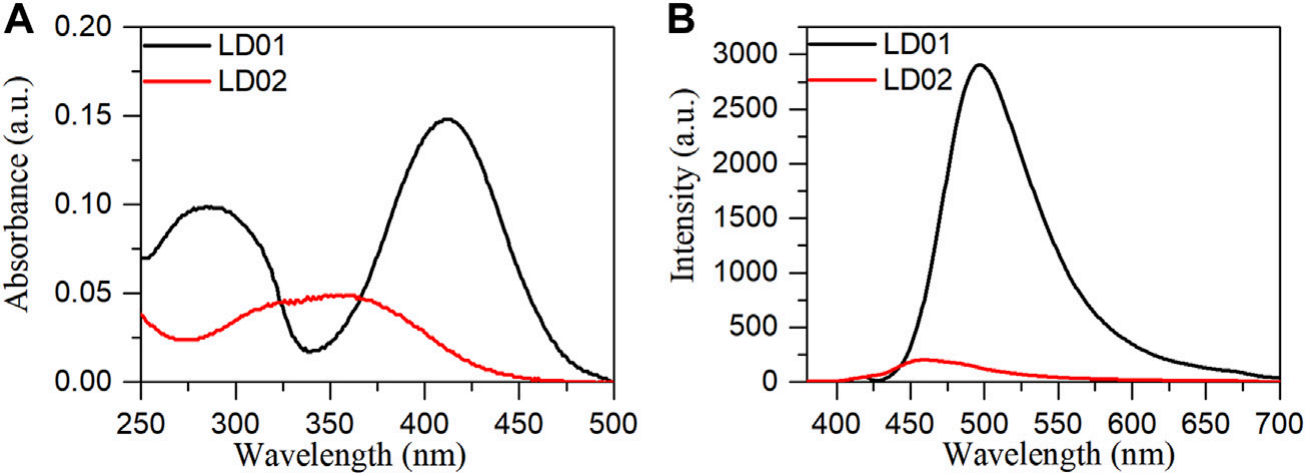
Absorption **(A)** and emission **(B)** spectra of **LD01** (λ_ex_ = 410 nm) and LD02 (λ_ex_ = 365 nm) in PBS buffer (10 mM, pH = 7.4).

**FIGURE 2:**
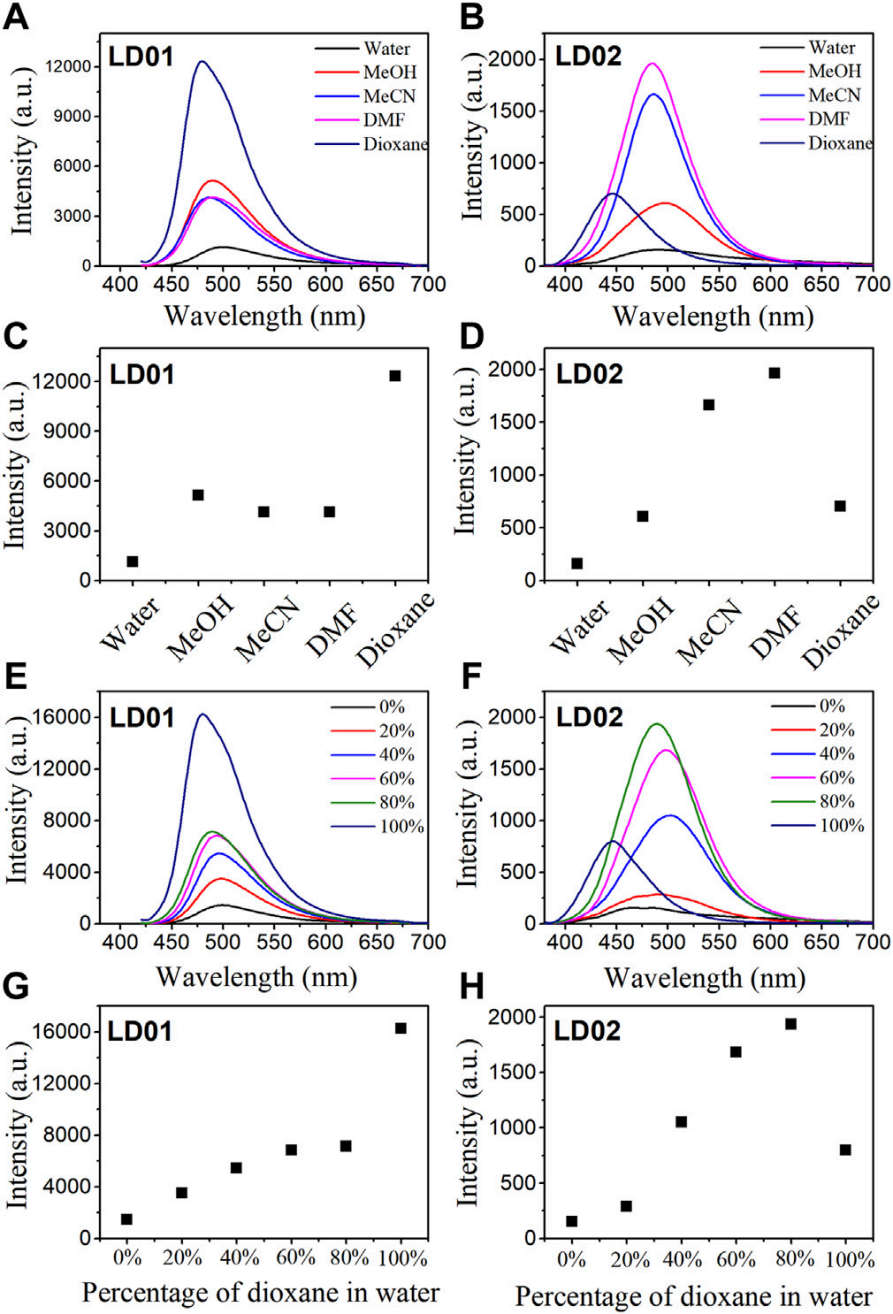
Fluorescence spectra of LD01 (λ_ex_ = 410 nm) and LD02 (λ_ex_ = 365 nm) in different solvents **(A, B)** and in mixed solvents of dioxane and water **(E, F)**. Scatterplots of the most intense fluorescence emission by LD01 **(C, G)** and LD02 **(D, H)** in **(A, B, E, and F)**, respectively.

**FIGURE 3:**
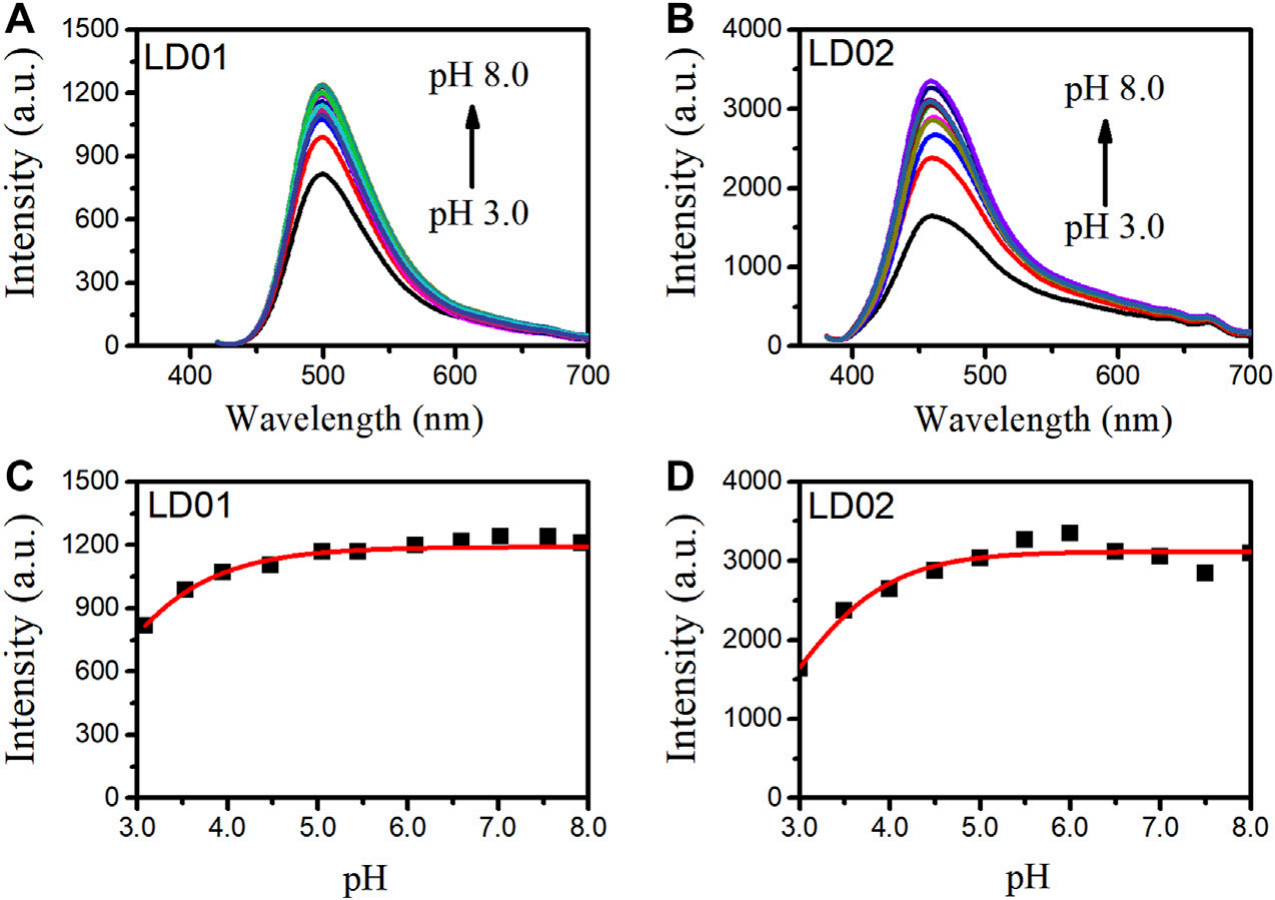
Fluorescence spectra of LD01 **(A)** (λ_ex_ = 410 nm) and LD02 **(B)** (λ_ex_ = 365 nm) in buffers of various pH. Scatterplots of the most intense fluorescence emission by LD01 **(C)** and LD02 **(D)** in **(A, B)**.

### Fluorescence Titration of LD01 and LD02 Toward α-Glucosidase

**FIGURE 4:**
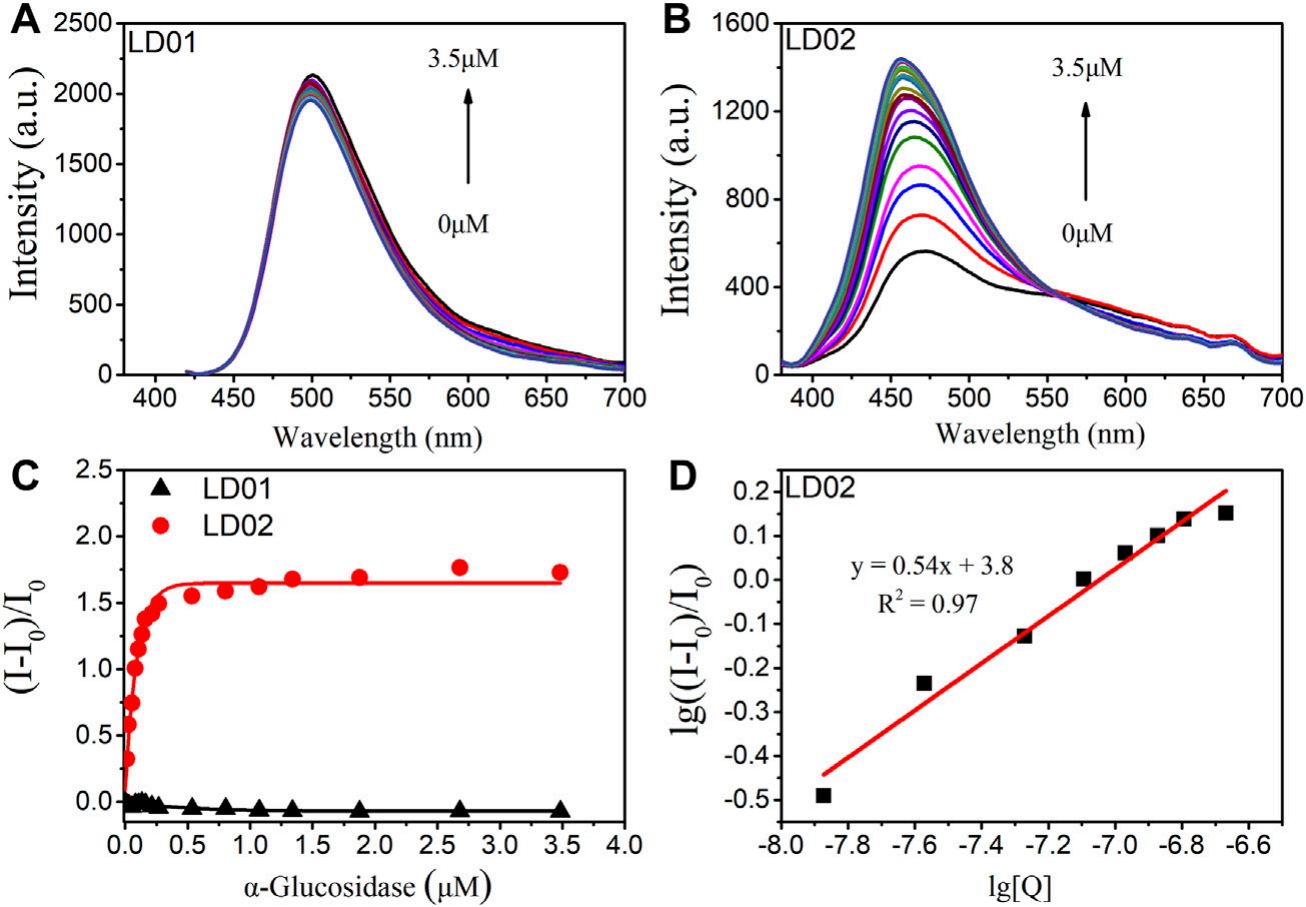
Fluorescence spectra of LD01 **(A)** and LD02 **(B)** (10 μM) in the presence of various concentrations of α-glucosidase (0–3.5 μM) in PBS buffer. **(C)** Changes in (I-I_0_/I_0_) of LD01 (λ_ex_ = 410 nm, λ_em_ = 500 nm) and LD02 (λ_ex_ = 365 nm, λ_em_ = 457 nm) on addition of α-glucosidase. **(D)** The linear regression line of lg ((I-I_0_)/I_0_) to lg [Q], [Q] is the concentration of α-glucosidase (mol/L).

To confirm the α-glucosidase-targeted binding interaction, fluorescence titrations were carried out by determining the emission spectra of LD01 and LD02 as a function of the concentration of α-glucosidase. As shown in Figure 4A, there are no obvious emission changes observed for LD01 after the gradual addition of α-glucosidase protein up to 3.5 μM. However, a significant enhancement of responsive fluorescence emission can be achieved for LD02 under the same experimental condition (Figures 4B, C). After the addition of 3.5 μM α-glucosidase, the maximum emission intensity increased by 2.7 times, and the emission maximum of LD02 was detected of a ∼7 nm blue shift from 457 to 450 nm, which suggests that the dimethylamine group in LD02 may locate in the binding pocket of relative lower polarity inside α-glucosidase than the aqueous surroundings. The binding constant and the rate of LD02 to α-glucosidase were obtained by the double logarithm regression curve as shown in Figure 4D. The value of log *K*_*a*_ of LD02 to α-glucosidase was calculated to be 3.8, and the binding ratio was approximately 0.5.

### Binding Selectivity of LD02 Toward α-Glucosidase

**FIGURE 5:**
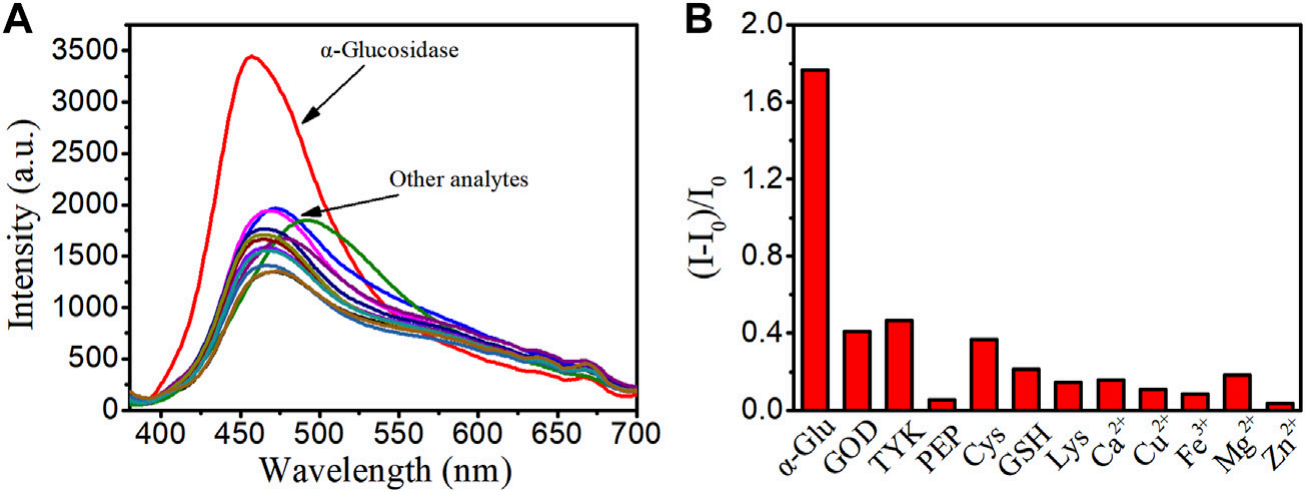
**(A)** Fluorescence spectra of LD02 (10 μM) in the presence of various species: α-glucosidase (α-Glu) (3.5 μM), GOD (3.5 μM), TKY (3.5 μM), PEP (3.5 μM), Cys (1 mM), GSH (1 mM), Lys (1 mM), Ca^2+^ (1 mM), Cu^2+^ (1 mM), Fe^3+^ (1 mM), Mg^2+^ (1 mM), and Zn^2+^ (1 mM) in PBS buffer. **(B)** The plot of (I-I_0_)/I_0_ in A (λ_ex_ = 365 nm, λ_em_ = 457 nm).

To verify the binding specificity of LD02 toward α-glucosidase, emission spectra of LD02 in the presence of α-glucosidase or various potential competitors such as other enzymes (including glucose oxidase (GOD), polyphenol oxidase (TKY), and pepsin (PEP)), and small molecules of interest (including Cys, GSH, Lys, Ca^2+^, Cu^2+^, Fe^3+^, Mg^2+^, and Zn^2+^), were collected in 10 mM PBS buffer (pH = 7.4) at room temperature. As shown in Figure 5, all potential competitors tested have no obvious effect on the emission band and intensity of LD02 compared with α-glucosidase, suggesting the highly specific selectivity of LD02 toward α-glucosidase.

### Competitive Fluorescence Titration

**FIGURE 6:**
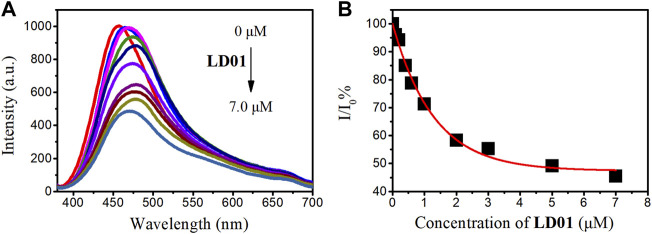
**(A)** Fluorescent emission spectra of α-glucosidase–LD02 binding complex (10 μM of LD02 with 3.5 μM of α-glucosidase, λ_ex_ = 365 nm) in the presence of various concentrations of LD01. **(B)** Scatterplots of the relative intensity I/I_0_ in A (λ_em_ = 457 nm).

For competitive fluorescence titration, different amounts of LD01 were added to a solution of α-glucosidase/LD02 complex. After the addition of LD01, it was found that the emission of α-glucosidase/LD02 complex decreased as a function of increasing the concentration of LD01, indicating LD01’s competitive binding to the same binding pocket as LD02 (Figure 6). The binding constant (*K*_*a*_) of LD01 to α-glucosidase was calculated, and the value of lg*K*_*a*_ was 5.2 for LD01, which means LD01 has a much stronger affinity for α-glucosidase than LD02 does. This result encouraged us to determine the inhibition efficiency of LD01 and LD02 against α-glucosidase.

### α-Glucosidase Inhibitory Effect of LD01 and LD02

**FIGURE 7:**
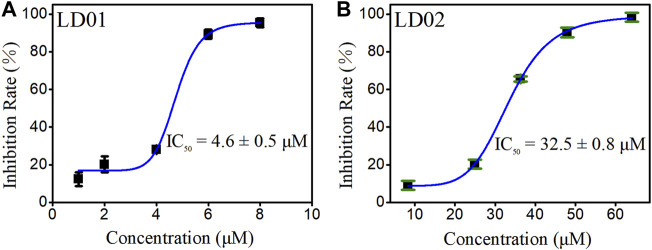
Plot of the inhibition rate of LD01 **(A)** and LD02 **(B)** against α-glucosidase concentration.

α-Glucosidase inhibitory efficiency of LD01 and LD02 was obtained from the inhibition rate vs. concentration curves of LD01 and LD02 (Figure 7). The results showed that coumarin derivatives LD01 and LD02 demonstrated excellent and potent inhibitory activity against α-glucosidase with the IC_50_ values of 4.6 ± 0.5 and 32.5 ± 0.8 μM, respectively, as compared to the standard acarbose (IC_50_ = 856.45 ± 5.60 μM). In particular, LD01 and LD02 have a relatively stronger inhibition effect against α-glucosidase than the coumarin derivatives that Xu et al. reported previously (Xu et al., 2019). In particular, the IC_50_ values of LD01 and LD02 have a positive correlation with the binding affinities observed from the fluorescence titration experiments, which indicates that our fluorescent probe LD02 can be used as an applicable tool helpful in the development and screening of pharmacologically active α-glucosidase inhibitors based on the candidate molecules’ binding strength.

## Conclusion

In summary, two environmentally sensitive fluorescence agents, LD01 and LD02, based on the conjugates of substituted coumarin and cinnamic acid, were designed and synthesized. Both LD01 and LD02’s fluorescent emissions show a polarity-dependent enhancement feature, as demonstrated in various solvents of different polarities, and the solvent mixture of dioxane and water. When coming to fluorescence titration, LD02 shows responsive emission changes featured with 2.7 times emission enhancement upon its binding to α-glucosidase. However, LD01 does not have such changes under the same experimental conditions as LD02 does. The competitive fluorescence titration of LD01 to the α-glucosidase/LD02 complex shows a gradually reduced emission as a function of increasing concentration of LD01, which suggests that LD01 can competitively bind to the same pocket of α-glucosidase targeted by LD02. The binding constants of LD01 and LD02 toward α-glucosidase were worked out by the fluorescence titration experiments, from which the values of lg *K*_*a*_ were found to be 5.2 and 3.8 for LD01 and LD02, respectively, and positively correlated with IC_50_ values (4.6 ± 0.5 and 32.5 ± 0.8 μM for LD01 and LD02, respectively) as observed from the enzyme inhibitory assays. This research not only lays out a new strategy for quantitative detection of α-glucosidase but also offered a potential tool for binding affinity evaluation between α-glucosidase and its inhibitors, which may provide guidance for developing new α-glucosidase inhibitors treating diabetes.

## Data Availability

The original contributions presented in the study are included in the article/Supplementary Material; further inquiries can be directed to the corresponding authors.
