# Supplementary material for: Responsive Fluorescent Coumarin–Cinnamic Acid Conjugates for α-Glucosidase Detection
Source: Front Chem. 2022 Jun 28;10:927624. doi: 10.3389/fchem.2022.927624 (PMC9273884; doi:10.3389/fchem.2022.927624)
Supplement: Supplementary file 1 [file DataSheet1.DOCX]

Supplementary Material

Responsive Fluorescent Coumarin-Cinnamic Acid Conjugates for α-Glucosidase Detection

Dong Luo^1†^, Xin Zhang^1†^, Xiaoying Li^1^, Yi-Yu Zhen^1^, Xingyi Zeng^1^, Zhuang Xiong^1*^, Yinghui Zhang^1,2*^, Hongguang Li^1,2 *^

^1^School of Biotechnology and Health Science, Wuyi University, Jiangmen 529020, China.

^2^Guangdong Provincial Key Laboratory of Large Animal Models for Biomedicine, South China Institute of Large Animal Models for Biomedicine, Wuyi University, Jiangmen 529020, China.

†**Dong Luo and Xin Zhang contributed equally and served as co-first authors.**

*** Correspondence:**Corresponding Authors
Hongguang Li, [wyuchemlihg@126.com](mailto:wyuchemlihg@126.com); Zhuang Xiong, [wyuchemxz@126.com](mailto:wyuchemxz@126.com); Yinghui Zhang, [wyuchemzyh@126.com](mailto:wyuchemzyh@126.com)

**Supplementary Figure 1.** ^1^H NMR spectrum of **LD01**. (500 MHz, DMSO-*d*_6_)

**Supplementary Figure 2.** ^13^C NMR spectrum of **LD01**. (125 MHz, DMSO-*d*_6_)


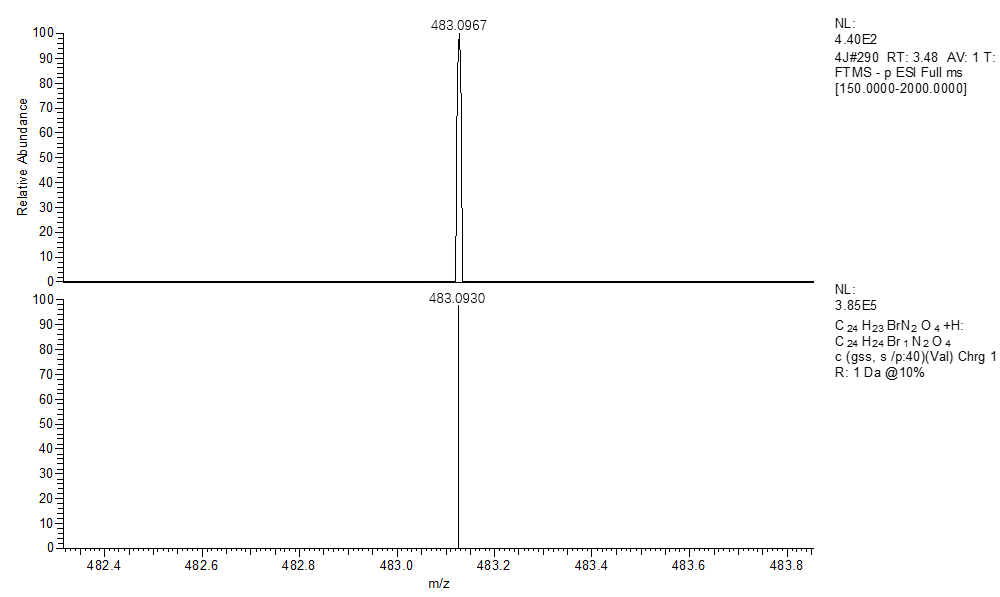


**Supplementary Figure 3.** HRMS (+ESI) spectrum of **LD01**. (m/z calcd. for C_24_H_23_BrN_2_O_4_ [M+H]^+^ 483.0930, found 483.0967.)

**Supplementary Figure 4.** ^1^H NMR spectrum of **LD02**. (500 MHz, DMSO-*d*_6_)

**Supplementary Figure 5.** ^13^C NMR spectrum of **LD02**. (125 MHz, DMSO-*d*_6_)


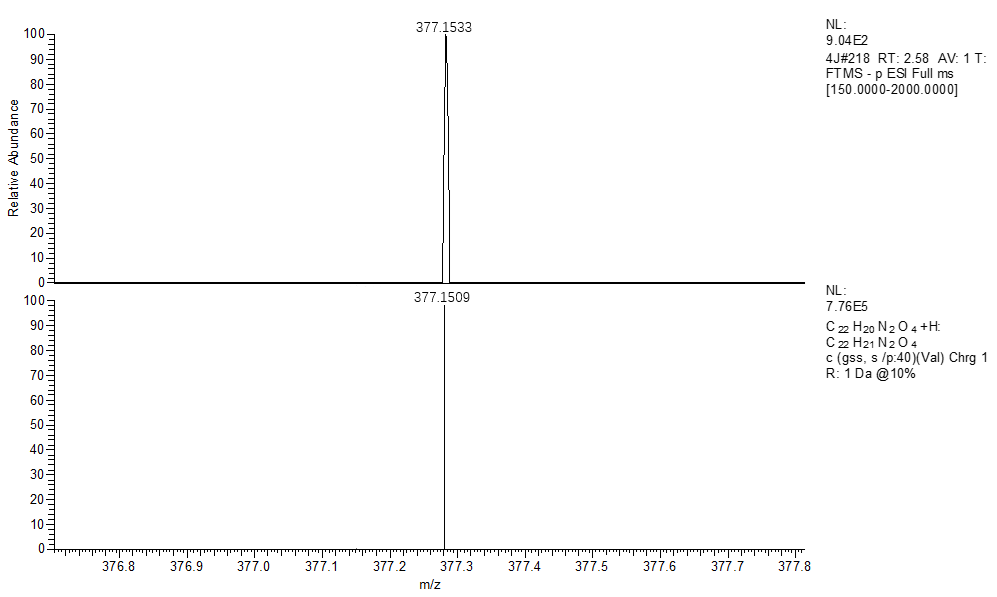


**Supplementary Figure 6.** HRMS (+ESI) spectrum of **LD02**. (HRMS (+ESI) calcd. for C_22_H_20_N_2_O_4_ [M+H]^+^ 377.1509, found 377.1533.)
